# Protection from COVID-19 disease in hamsters vaccinated with subunit SARS-CoV-2 S1 mucosal vaccines adjuvanted with different adjuvants

**DOI:** 10.3389/fimmu.2023.1154496

**Published:** 2023-03-20

**Authors:** Yongjun Sui, Hanne Andersen, Jianping Li, Tanya Hoang, Yonas Bekele, Swagata Kar, Mark G. Lewis, Jay A. Berzofsky

**Affiliations:** ^1^ Vaccine Branch, Center for Cancer Research, National Cancer Institute, National Institutes of Health (NIH), Bethesda, MD, United States; ^2^ BIOQUAL Inc., Rockville, MD, United States

**Keywords:** SARS-CoV-2, COVID-19, intranasal vaccine, mucosal adjuvant, hamster model

## Abstract

**Introduction:**

Adjuvant plays an important role in directing the immune responses induced by vaccines. In previous studies, we have shown that a mucosal SARS-CoV-2 S1 subunit vaccine adjuvanted with a combination of CpG, Poly I:C and IL-15 (named CP15) induced effective mucosal and systemic immunity and conferred nearly sterile protection against SARS-CoV-2 viral replication in macaque models.

**Methods:**

In this study, we used a hamster model, which mimics the human scenario and reliably exhibits severe SARS-CoV-2 disease similar to hospitalized patients, to investigate the protection efficacy of the vaccines against COVID-19 disease. We compared the weight loss, viral loads (VLs), and clinical observation scores of three different vaccine regimens. All three regimens consisted of priming/boosting with S1 subunit vaccines, but adjuvanted with alum and/or CP15 administrated by either intramuscular (IM) or intranasal (IN) routes: Group 1 was adjuvanted with alum/alum administrated IM/IM; Group 2 was alum-IM/CP15-IN; and Group 3 was CP15-IM/CP15-IN.

**Results:**

After challenge with SARS-CoV-2 WA strain, we found that the alum/CP15 group showed best protection against weight loss, while the CP15 group demonstrated best reduction of oral SARS-CoV-2 VLs, suggesting that the protection profiles were different. Sex differences for VL and clinical scores were observed. Humoral immunity was induced but not correlated with protection. Moreover, S1-specific binding antibody titers against beta, omicron BA.1, and BA.2 variants showed 2.6-, 4.9- and 2.8- fold reduction, respectively, compared to the Wuhan strain.

**Discussion:**

Overall, the data suggested that adjuvants in subunit vaccines determine the protection profiles after SARS-CoV-2 infection and that nasal/oral mucosal immunization can protect against systemic COVID-19 disease.

## Introduction

Subunit vaccines have the advantage of being safe and stable under refrigeration conditions. However, poor immunogenicity imposes the use of adjuvants in the development of this type of vaccines. Adjuvants provide help to a given vaccine by enhancing the immune responses to reduce the amounts of antigens or the doses of boosters used, and to increase immunogenicity by marshalling an innate immune response. Moreover, adjuvants are also able to modulate or direct the types of immune responses induced by vaccines, for example alter humoral or cellular, Th1 or Th2, or modify the breath, specificity, affinity or longevity of the responses (1–3), and thus qualitatively affect the mechanism and type of protection. Among the various adjuvants used in human vaccines, aluminum salts (Alum) are the most widely used, which have been approved for a list of licensed human vaccines including haemophilus influenzae type b (Hib), hepatitis A and B virus, tetanus, meningococcal, human papillomavirus, and diphtheria (4, 5). In SARS-CoV-2, alum adjuvanted vaccines have been approved recently (6, 7). As SARS-CoV-2, the causative agent of COVID-19, mainly infects the host through the respiratory tract, it is important to develop mucosal vaccines that could prevent the establishment of infection at the portal of entry (8, 9). Though alum is efficacious in inducing protective immunity, it cannot be used as a mucosal adjuvant. Besides alum, a set of molecular adjuvants including toll-like receptor (TLR) ligands are currently used in vaccine development (10–12). We have utilized an adjuvant combination, which is composed of TLR agonists CpG and Poly I:C, and the cytokine IL-15, in HIV vaccine studies, and found that the combination of adjuvants (which we call CP15) can effectively facilitate the induction of innate and adaptive immunity to control the transmission of simian HIV in macaques and to protect against the CD4^+^ T cell loss after the infection of SIVmac251 (13, 14). While both TLR agonists and IL-15 prompted potent T cells responses (15, 16), the combination of TLR agonists and IL-15 had synergistic effect for driving adaptive responses (13). Moreover, CP15 could be a good candidate for a mucosal adjuvant, as it could be delivered both intramuscularly and intranasally (17, 18). The CP15 adjuvanted vaccines induced effective immune responses and conferred nearly sterile protection against SARS-CoV-2 viral replication in rhesus macaque models (17, 18).

The macaque model, which presents with only a mild clinical course of COVID-19 disease and transient viral load, cannot be used to evaluate the protective efficacy of the adjuvanted subunit vaccine against COVID-19 disease. Syrian golden hamsters, whose ACE2 receptor is similar to that of humans, are naturally permissive to SARS-CoV-2 infection. The clinical, virologic, and pathological manifestations of the disease in golden Syrian hamster models resemble a much more severe form of human COVID-19 disease after SARS-CoV-2 infection (19, 20). Herein, we used the golden Syrian hamster model to compare the protective efficacy of SARS-CoV-2 S1 subunit vaccine adjuvanted with either Alum or CP15 or both, delivered systemically or mucosally. We included three vaccination regimens, which consisted of priming/boosting with S1 subunit vaccines, but adjuvanted with alum and/or CP15 administrated by either intramuscular (IM) or intranasal (IN) routes: Group 1 received the S1 protein adjuvanted with alum/alum administrated IM/IM; Group 2 received S1 in alum as prime IM/CP15-IN as boost; and Group 3 received S1 with CP15/CP15-at both IM prime and IN boost. After challenge with SARS-CoV-2, we found that the alum/CP15 group showed significant protection against weight loss, while the CP15/CP15 group demonstrated significant reduction of oral SARS-CoV-2 VLs. Interestingly, we found that humoral immunity in serum was not correlated with weight loss and viral loads, suggesting that other factors might be involved in protection.

## Results

### Alum/CP group showed significant protection against weight loss after SARS-CoV-2 infection

**Figure 1:**
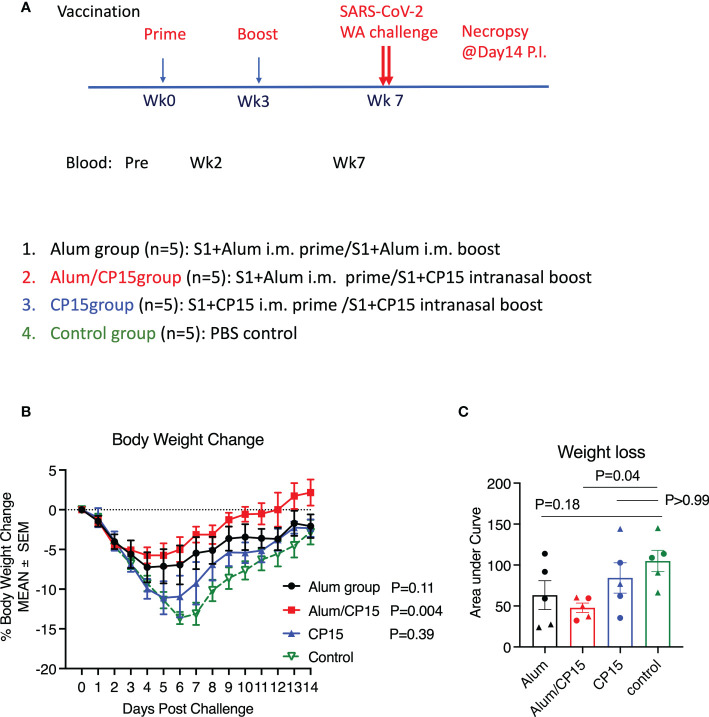
The Alum/CP15 group, which had been vaccinated with S1 protein adjuvanted with alum/CP15, showed significant protection against weight loss after challenge with SARS-Cov-2 WA strain in Syrian golden hamsters. **(A)**. Schematic diagram of vaccination/challenge schedules, and vaccination groups. **(B, C)**. Kinetics of body weight loss **(B)** and the area under curve of body weight loss **(C)** in hamsters after challenge with SARS-CoV-2 WA strain. N=5 for each group, and each dot in **(C)** represents one animal. Two-way ANOVA and Kruskal-Wallis tests with Dunn’s multiple comparison corrections were used to compare between the vaccinated groups and the control group. Mean ± SEM are shown.

We have used CP15, a combination of CpG, poly I:C and IL-15, as an adjuvant to elicit robust immune responses, and vaccinating with SARS-CoV-2 S1 protein in this adjuvant conferred nearly sterile protection against SARS-CoV-2 viral replication in macaque models (17, 18). To evaluate the protective effect of the CP15-adjuvanted SARS-CoV-2 subunit vaccine against COVID-19 disease, we immunized three groups of hamsters, 5 animals per group, with SARS-CoV-2 S1 (Wuhan strain) as antigen but with different combinations of adjuvants (**Figure 1A**). Group 1 (Alum) was a systemic vaccine, in which the animals received two doses of intramuscularly (IM) administrated S1 protein adjuvanted with alum. This group served as control group, as alum is one of the most widely used adjuvants in various licensed vaccines including SARS-CoV-2 vaccine (6, 7). Group 2 (Alum/CP15) and group 3 (CP15) were mucosal vaccines, in which the animals received S1 adjuvanted with alum IM (for group 2) or adjuvanted with CP15 (for group 3), and then both groups were intranasally (IN) boosted with S1 adjuvanted with CP15. The hamsters were immunized at Week-0 and Week-3. Four weeks after the second dose, all the vaccinated groups, as well as a naïve (unvaccinated) group (group 4), were intranasally challenged with 3X10^4 pfu SARS-CoV-2 Washington (WA/2020) strain (**Figure 1A**).

Upon SARS-CoV-2 infection, the naive group demonstrated rapid weight loss starting from Day 2. The weight loss trend continued until Day 7 (about 15% weight loss), and then gradually recovered (**Figure 1B**). Compared to the naïve control group, group 2 (Alum/CP15) showed the best protection against weight loss (P=0.004), while group 1 (Alum) showed a trend of protection (P=0.11, **Figure 1B**). There was no significant protection in the CP15 only group (P=0.39, **Figure 1B**). The area under curve (AUC) of weight loss confirmed that only group 2 (Alum/CP15) had significant reduction of weight loss compared to the naïve control group (p=0.04, **Figure 1C**).

As both female and male animals were included in the study, we assessed whether sex of the animals played a role in protection against weight loss after SARS-CoV-2 WA strain infection. Even with only 2 or 3 animals per group, we found that both females and males in group 2 (Alum/CP15), and male animals in group 1 (Alum), demonstrated trends of protection against weight loss, while the rest of them did not (**SFigure 1**). If all the animals were considered, it seemed that sex did not affect weight loss (**SFigure 1**).

### Significant reduction of oral viral loads after SARS-CoV-2 infection in hamsters immunized with the CP15 adjuvant

**Figure 2:**
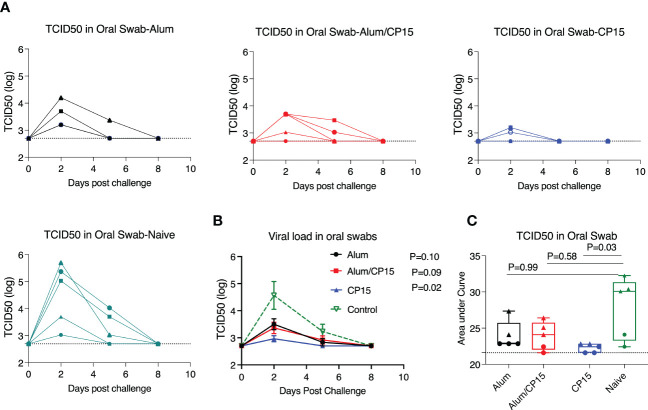
The CP15 group showed significant oral swab viral load reduction after challenge with SARS-Cov-2 WA strain in Syrian golden hamsters. After challenge with SARS-CoV-2 WA strain, oral swabs from each hamster were collected at different time points and viral loads were measured as TCID50. **(A, B)**. Oral swab viral loads of each hamster from different groups **(A)** and the summary (means) of each group **(B)** are shown. **(C)** The area under the curve of oral swab viral loads from each group are shown. N=5 for each group, and each symbol represents one animal in **(A)**. The dots denote female, while the triangles denote male in **(C)**. Two-way ANOVA and Kruskal-Wallis tests with Dunn’s multiple comparison corrections were used to compare between the vaccinated groups and the control group. Dashed line shows the detection limit. Mean ± SEM are shown.

After SARS-CoV-2 infection, the viral loads (VL) in oral swabs of the hamsters were measured at Day 2, 5, and 8 post challenge. All five naïve animals were infected. The VLs peaked at Day 2, declined at Day 5, and cleared at Day 8 (**Figure 2A**). All the vaccinated groups showed a similar trend, but with lower VLs. Notably, the 3 infected animals in group 3 (CP15) cleared their VL at Day 5 (**Figure 2A**). Specifically, one animal in group 2 (Alum/CP15) and two animals in group 3 (CP15) had no detectable VLs during the whole observation period (**Figure 2A**). Compared to the naïve control group, group 3 (CP15) demonstrated significantly reduced VLs (P=0.02 for kinetics and P=0.03 for AUC), while group 1 (Alum, P=0.10 for kinetics and P=0.99 for AUC) and group 2 (Alum/CP15, P=0.09 for kinetics and P=0.58 for AUC)) only showed trends or not significant reduction of VLs (**Figures 2B, C**).

Usually, when the VLs are controlled, the infected hosts will have less severe disease. However, here we observed that group 3 (CP15), which had the lowest VLs among the groups, suffered the most severe weight loss. This is consistent with observations that VLs in SARS-CoV-2 infected patients did not match with the severity of COVID-19 disease, suggesting that the disease severity was not determined only by the VLs, but other factors also played important roles (21).

Fourteen days after the viral infection, we measured the SARS-CoV-2 viral load in the lung parenchyma of the hamsters. We found that in the vaccinated groups, no viral load was detected in the lung of the group 2 animals, while group 1 and group 3 each had one animal showing detectable viral load. In contrast, the naïve controls had viral loads detected in the lungs of 4 out of 5 animals (**SFigure 2**).

### Sex difference was present for viral load, clinical observations, but not for weight loss

**Figure 3:**
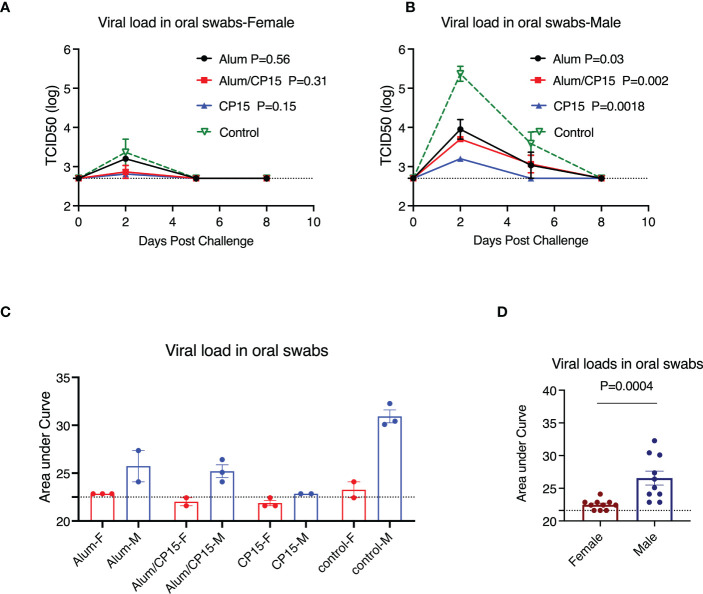
Sex difference in viral load reduction after challenge with SARS-CoV-2 WA strain in Syrian golden hamsters. **(A, B)**. Kinetics of viral load reduction in vaccinated and naïve female **(A)** and male **(B)** hamsters. **(C, D)**. Comparisons of area under curve of viral load reduction between female and male hamsters after SARS-CoV-2 WA strain infection. Each symbol represents one animal in **(C, D)**. Two-way ANOVA analyses were used to compare the vaccinated groups and control group. Mann-Whitney test was used to compare the females and males. Dashed lines show the detection limit. Mean ± SEM are shown.

VLs in females and males from each group were compared separately. We found that female animals effectively controlled VLs, including the naïve controls. Virus was present only at Day 2 post challenge for all the females (**Figure 3A**). As a result, none of the females in the vaccinated groups differed significantly from the naïve control group (**Figure 3A**). For males, the naïve control group had high VLs, which peaked at Day 2 post challenge, and declined at Day 5 (**Figure 3B**). Compared to the males in the naïve control group, all vaccinated males, regardless of regimen, had significantly lower VLs (**Figure 3B**). Group 1 (Alum) and group 2 (Alum/CP15) had a pattern similar to that of the naïve control group, while virus was only detected in group 3 (CP15) on Day 2 post infection (**Figure 3B**). Overall, females had much lower VLs than males did (**Figures 3C, D**).

**Figure 4:**
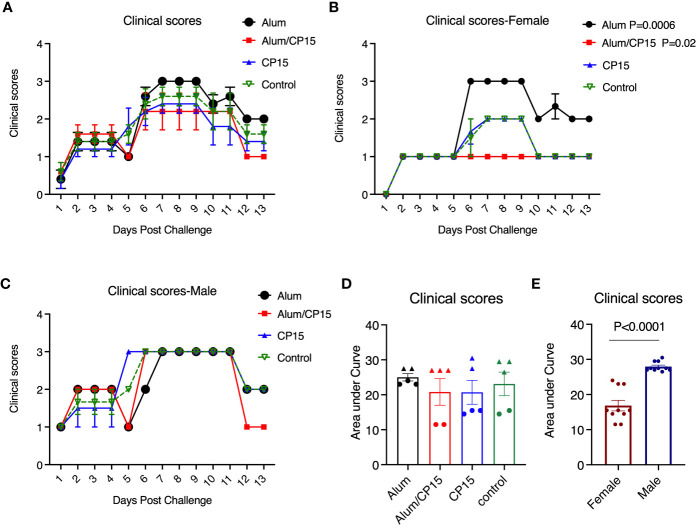
Clinical scores of the hamsters after SARS-CoV-2 Washington strain challenge. **(A–C)**. After SARS-CoV-2 Washington strain infection, animals were monitored, and clinical scores were given each day. The clinical score was based on the observation whether ruffled fur and/or hunched back was present in the animals: milder ruffled fur =1; ruffled fur= 2, and hunched back=1, and if none of them was present the animal will be given a score of zero. The clinical score was calculated based the sum of ruffled fur and hunched back scores. Kinetics of clinical score changes in all the animals **(A)**, females **(B)**, and males **(C)** of difference groups. **(D, E)**. Comparisons of area under curve of clinical score changes in females and males. N=5 for each group. The triangles denote males, and the dots denote females in **(D)**. Each dot represents one animal in **(E)**. Two-way ANOVA and Mann-Whitney analysis were used to compare between the vaccinated groups and the control group. Mean ± SEM are shown.

We further evaluated another independent clinical observation: the clinical scores, which was calculated by the sum of scores of ruffled fur and hunched back (Clinical scores in **Figure 4**, are dissected into their subcomponents in **SFigures 3, 4**). If both sexes were included, no significant changes were found between the vaccinated groups and the naive group. In female animals, the clinical scores in group 2 (Alum/CP15) were lower, and in group 1 (Alum) were higher than those of the naïve group (**Figure 4B**). The latter was an alerting sign that warrants further investigation in future studies. Interestingly, we also found that females had lower scores for clinical parameters than those of males (**Figure 4E**), which was consistent with the COVID-19 symptoms seen in real-world settings (22, 23).

### Humoral immune responses induced by S1 subunit vaccines did not correlate with weight loss or VLs

**Figure 5:**
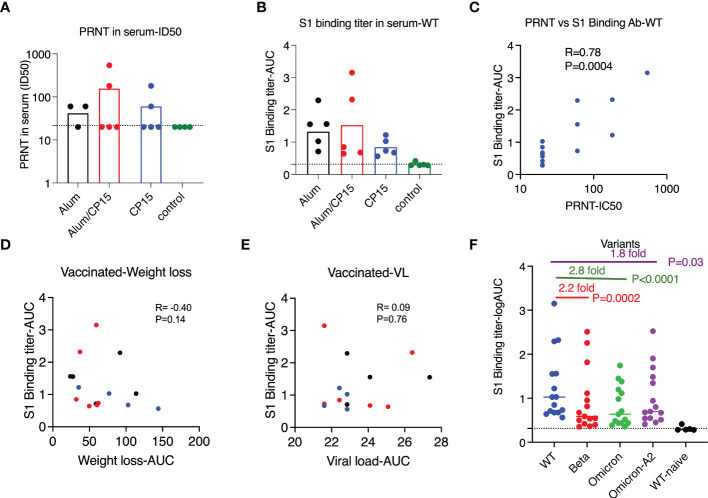
Vaccine-induced humoral immunity did not correlate with protection after challenge with SARS-CoV-2 WA strain in Syrian golden hamsters. **(A, B)**. PRNT titers **(A)** and S1-specific binding antibody titers **(B)** against SARS-CoV-2 WA/Wuhan strain in the serum of the vaccinated and naïve animals (4 weeks after the second vaccination). **(C)** S1-specific binding antibody correlated with PRNT titers. **(D, E)**. S1-specific binding antibody titers did not correlate with weight loss **(D)** or viral load reduction **(E)** of the vaccinated animals. Spearman analyses were used for the correlations. **(F)**. S1-specific binding antibody titers against S1 of SARS-CoV-2 wild type (Wuhan) strain, beta, Omicron, and Omicron A.2 variants. Each symbol represents one animal. Non-parametric one-way ANOVA analyses with Dunn’s multiple comparison correction was used to compare the titers against wild type and the variants. Dashed line shows the titers of the naïve animals. Means ± SEM are shown.

As it is important to find the correlates of protection against SARS-CoV-2 infection and COVID-19 disease, we assessed whether humoral immune responses induced by vaccines contributed to the control of VLs, weight loss and other clinical manifestations. After two doses of vaccines, only 2 animals in each vaccinated group had detectable neutralizing antibody titers measured by live virus plaque reduction neutralization test (PRNT) titers (**Figure 5A**). We did not find any correlations between PRNT neutralization titers and weight loss (Spearman’s R= - 0.3, P=0.14) or VLs (R= -0.12, P=0.70). The rest of the clinical manifestations, such as clinical scores, as well as its subcomponents ruffled fur score and hunched back score, did not correlate with PRNT titers either (Spearman’s R= - 0.32, P=0.27; R= - 0.28, P=0.36; PR= -0.36, P=0.22 respectively).

All the vaccinated animals had detectable binding antibody titers against SARS-CoV-2 S1, and there was no significant difference among the vaccinated groups (**Figure 5B**). Consistent with the studies in the field, the binding antibody titer was positively correlated with PRNT (**Figure 5C**, R=0.78, P=0.004). We did not find sex differences in the binding antibody titers against wild type S1(Mann-Whitney P=0.58). When evaluating the contributions of S1 binding antibody to the control of weight loss (R= - 0.40, P=0.14) and VLs (R= 0.09, P=0.76), we did not observe any significant associations (**Figures 5D–E**), which was consistent with the PRNT data. The vaccine-induced S1 binding antibody also had cross-reactivity with other SARS-CoV-2 variants. However, compared to wild type, the median titers against S1 from beta, Omicron, and Omicron A.2 were 2.2-, 2.8, and 1.8-fold lower (**Figure 5F**).

**Figure 6:**
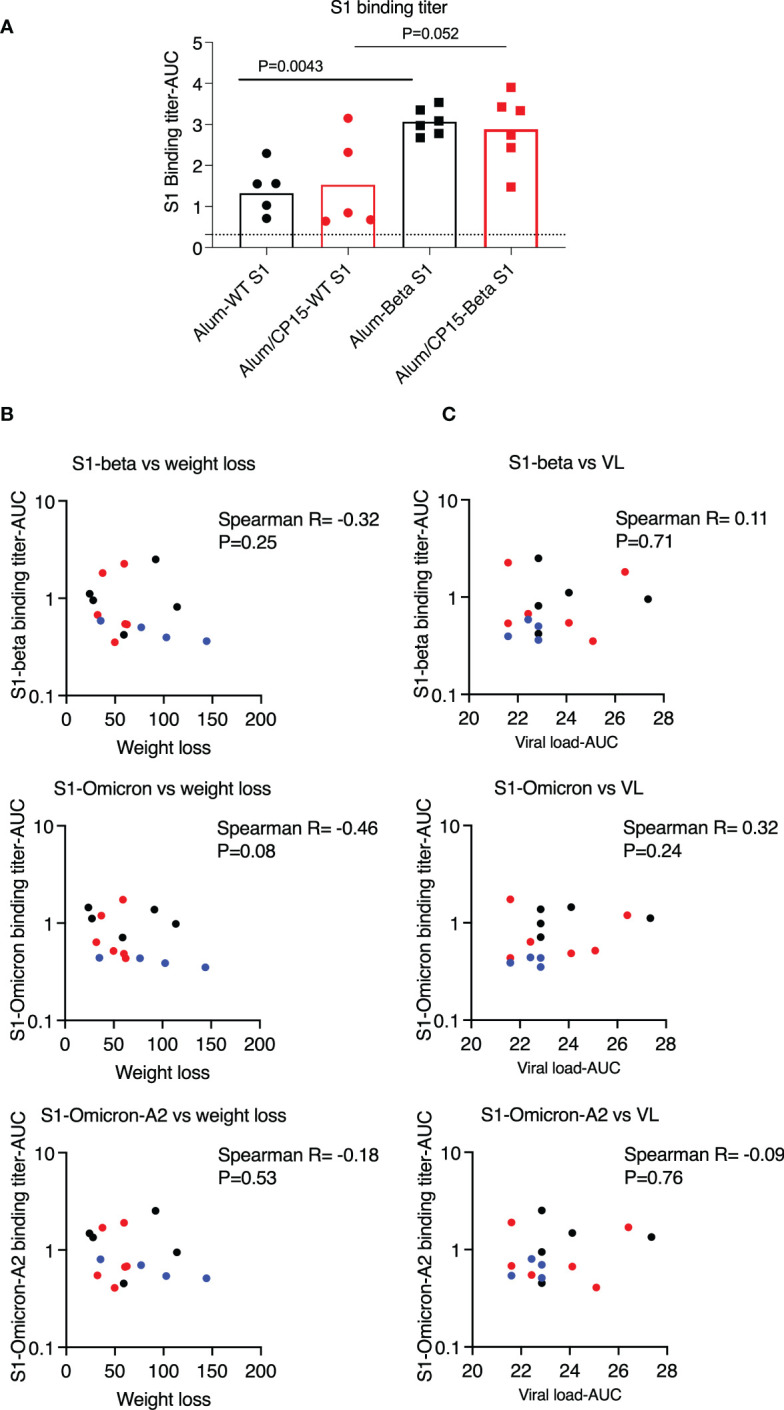
Binding antibody titers against S1 variants, which were lower than those of vaccinated with beta variant S1, did not correlate with weight loss or viral loads (VL) in the hamsters after SARS-CoV-2 Washington strain challenge. **(A)**. To compare the humoral immune responses, we measured the serum binding antibody titers against Wuhan (Wild type, WT) strain from both this cohort, as well as another cohort of hamsters, which were vaccinated with the same regimen as group1&2 of this cohort except the S1 protein was substituted by S1 protein from the beta variant. Serum binding antibody titers against S1 (WT) were lower in this cohort, which were vaccinated with S1 from WT, than those of animals vaccinated with the beta variant S1. **(B, C)**. Binding antibody titers against S1 variants (4 weeks after the second vaccination) did not correlate with weight loss **(B)** or VL **(C)**. Spearman analyses were used for the correlations. .

When comparing the serum S1 binding antibody titers with another cohort, where hamsters were vaccinated with the same regimen as group 1 (n=6) and group 2 (n=6) except the S1 protein was substituted by S1 protein from the beta variant, we found that the titers were much lower in the cohort vaccinated with wild type S1 than those of hamsters vaccinated with beta variant S1 (**Figure 6A**). In the case of low humoral response, we found that for the S1 binding antibody titers, there were no difference between females and males and the binding antibody titers against S1 variants did not correlate with weight loss or VLs after SARS-CoV-2 infection (**Figures 6B, C**).

## Discussion

Adjuvant affects vaccine efficacy by increasing and/or modulating the types of immune responses induced by vaccines. In this study, we compared the protective efficacy against SARS-CoV-2 WA strain in three groups of hamsters vaccinated with S1 subunit vaccine with different adjuvant combinations. Upon SARS-CoV-2 WA strain challenge, the two groups that received the IN administered vaccine adjuvanted with CP15 as a booster demonstrated significant protection against weight loss or viral replication, suggesting the advantage of mucosal vaccine delivery. As the choice of the adjuvant for subunit vaccines is a key factor in protection, the development of mucosal vaccine adjuvants is especially critical. Mucosal adjuvants act not only as a delivery vehicle, which helps the antigen to cross the mucosal membranes and to be taken up by dendritic cells, but also, as an immunostimulatory agent, which facilitates the induction of immune responses in the mucosal-associated lymphoid tissues. Here we used DOTAP as a delivery vehicle so that the vaccine complex forms a nanoparticle; we also included TLR agonists CpG and polyI:C, as well as IL-15, to stimulate both innate and adaptive immunity. Overall, the CP15 combination is a potent candidate as a mucosal adjuvant.

We found that group 2 (Alum/CP15) showed significant protection against weight loss, whereas group 3 (CP15) had the most significant VL reduction. Clearly, the VL reduction here did not result in the alleviation of the COVID-19 disease, as assessed by protection against weight loss or reduced clinical scores. This was inconsistent with an early systemic review including 60 studies, which found that high SARS-CoV-2 VL was an independent predictor of disease severity and mortality in the majority of the studies on SARS-CoV-2 infected individuals (24). However, our study was consistent with a later review with 34 studies, which showed that the relationship between COVID-19 severity and VL was inconclusive, as a similar number of studies either supported or opposed this association (21). These data show that a human vaccine needs to be tested for protection against both viral load and COVID-19 disease.

The Syrian golden hamster is a well-characterized SARS-CoV-2 infection animal model. The clinical disease includes rapid weight loss accompanied by high VL in the upper and lower respiratory tract, as well as other clinical symptoms including rapid breathing, ruffled fur, and hunched back (19, 20, 25). In this study, we found that the humoral immune responses did not correlate with any of the viral or clinical manifestations, including oral VLs, weight loss and clinical scores, after SARS-CoV-2 infection. Recent studies suggested that antibody, T cell, and innate immunity all contribute to COVID-19 protection (26–28). Specifically, in the presence of high antibody titers, humoral immunity was correlated with protection, while in the case of low titers of antibody, T cells and innate immunity were the major factors controlling the virus (27). Indeed, we found that the S1 binding and live virus neutralization antibody titers were lower in this study compared to another study of immunization with SARS-CoV-2 beta variant S1 with the same adjuvant combination. Due to the lack of reagents recognizing hamster T cell markers, it was not possible to measure viral-specific-T cell responses. However, in the previous macaque studies, we found that both alum and alum/CP15-containing vaccines elicited vigorous T cell immunity in the periphery as well as in the lung (17). It is well known that alum induces a mixture of Th1 and Th2 responses in humans and macaques, while more restricted Th2 responses were elicited in mice (29). Thus, as for the type of T cell responses induced, it was possible that in this hamster study, the T cell responses elicited by the CP15-adjuvanted vaccine were more diverse and potent than those elicited by the alum vaccine. In fact, in a mouse model, we confirmed the hypothesis that an Alum/CP15-adjuvanted vaccine induced more potent T cell responses than Alum alone (Li et al, manuscript in preparation). One advantage of T cell-mediated immunity is that it tends to be long-lasting and most importantly, less prone to be affected by mutations of variants that tend to be limited to the spike protein (30). Moreover, after stimulation with viral mimics, lung cells from macaques that received CP15-adjuvanted vaccines produced higher type I interferon than those from naïve macaques (17). In two recent studies, the induction of trained immunity was observed transiently (20 days) or up to 83 days presented in the peripheral monocytes after the individuals received BNT162b2 mRNA and ChAdOx1 nCoV-19 (AZD1222) vaccines (31, 32). We found that CD16^+^ monocytes in the lung bronchoalveolar lavage (BAL) fluid were increased after the macaques received the same vaccination adjuvanted with CP15 as the hamsters (17). In line with this, BNT162b2 mRNA booster vaccination induced higher frequency of CD14^+^CD16^+^ inflammatory monocytes (33). This non-classical monocyte population was decreased in SARS-CoV-2-infected patients compared to those in the healthy controls or vaccinated individuals, suggesting that they might contribute to viral control (34).

In summary, alum- and CP15- adjuvanted mucosal vaccines had different protection profiles in the hamster model of COVID-19. The combination of both adjuvants mediated protection against COVID-19 disease, not just viral replication, even in the absence of high titers of binding and neutralization antibodies. Further, the lack of correlation between VL and clinical parameters suggests that for human vaccine studies, it is important to measure both VL and disease parameters independently. In addition, vaccine trials should take into account differences in vaccine responses by males and females and adequately power the trials for both sexes.

## Material and methods

### Animals and study design

Ten male and 10 female Syrian golden hamsters (Envigo), 8–10 weeks old, were housed at BIOQUAL, Inc. (Rockville, MD). All animal studies were approved by the Institutional Animal Care and Use Committee of BIOQUAL, Inc. and conducted in compliance with all relevant regulations. The hamsters were grouped randomly into 4 groups, and each group contained 2-3 females and males. The animals were administered the vaccinations as indicated in **Figure 1A**. Briefly, Group 1 (Alum) animals were primed and boosted i.m. with S1+alum; group 2 (Alum/CP15) animals were primed i.m. with S1+alum, and boosted i.n with S1+CP15; group 3 (CP15) animals were primed i.m. and boosted i.n. both with S1+CP15; group 4 received only PBS, and served as a control group. The components and the dose of the vaccine were as listed below: 20 μg per dose of SARS-CoV-2 S1 protein (WA strain, catalog: 40591-V08H from Sino Biological) was used as antigen for all the vaccinations; 10 μl of aluminum phosphate gel (alum: Catalog: vac-phos-250, *In vivo*Gen) was used as alum adjuvant. CP15 adjuvant was composed of 20 μg per dose of D-type CpG oligodeoxynucleotide (Catalog: vac-1826-1, *In vivo*Gen), 40 μg per dose of Poly I:C (Catalog # vac-pic, *In vivo*Gen), and 20 μg per dose of recombinant murine IL-15 (Catalog: 210-15, PeproTech). The S1 protein and CP15 were mixed with 20 μl of DOTAP (Cat. No. 11 811 177 001, Roche Inc.). For the IN-dosing procedures, the hamsters were sedated with Ketamine(80mg/kg)/Xylazine(5mg/kg). The dose volume for the vaccines was 50 uL into each nare so 100 uL total prepared vaccine per hamster *via* the IN route. Using this procedure (sedation and size of the inoculum), the dosing material would likely have penetrated further into the respiratory tract, getting to the lungs.

To compare the humoral immune responses, we measured the serum binding antibody titers against Wuhan (Wild type, WT) strain from another cohort of hamsters, which were vaccinated with the same regimen as group 1 (n=6) and group 2 (n=6) of this cohort except the S1 protein was substituted by S1 protein from the beta variant.

### SARS-CoV-2 WA strain challenge and monitoring of weight loss and clinical scores

Four weeks after the last vaccination, all animals were challenged with 1.99 × 10^4^ TCID50, which was equivalent to 3 × 10^4^ plaque-forming units (PFU) of SARS-CoV-2/USA-WA1/2020 P4 Animal Challenge Stock (Lot no. 70038893, NR-53780, BEI Resources) intranasally (50 μl/nare). Body weights were measured before and after the viral challenge. Clinical scores were monitored for each animal daily and blinded upon challenge for 2 weeks. The weight loss and clinical scores were independent parameters. The clinical scores were based on the observation whether ruffled fur and/or hunched back was present in the animals. The scores were given based on the presence of the following: milder ruffed fur =1; ruffled fur= 2, and hunched back=1, and if none of them was present the animal will be given a score of zero. The clinical score was calculated based the sum of ruffled fur and hunched back scores, which were independent of each other, but not independent of clinical score. We assessed the overall clinical score and dissected it into its two components of ruffled fur and hunched back as well.

### S1-specific binding antibody titer measurement

The S1-specific binding antibody titer was measured using ELISA assays as described before (6). 100 ng/well of the SARS-CoV-2 spike S1-His recombinant proteins (WA strain, catalog: 40591-V08H; beta variant, catalog: 40591-V08H10; Omicron variant, 40591-V08H41; Omicron A.2 variant, Cat: 40591-V08H43; all from Sino Biological) were coated in high-binding 96-well plates (Santa Cruz Biotechnology) overnight at 4°C. After four washes, the plates were blocked 1 hr with 300 μL of 1X PBS with 2% sodium casein. Serum samples (with a series of 4-fold dilutions starting from 1:100) were applied in duplicate to the plates. After 1 hr of incubation at room temperature and four washes, anti-hamster IgG -HRP conjugate (1:10,000 dilution, ThermoFisher) was added and incubated for 1 hr at room temperature. TMB substrate was added following 4 washes as described before. Areas under the curve were calculated using GraphPad Prism 9 software.

### Plaque reduction neutralization test (PRNT)

The PRNT assay was used to measure neutralization antibody titers as described before (6). Three-fold serial dilutions of serum samples starting from 1:20, and up to a final dilution of 1: 4860, were incubated with 30 pfu of SARS-CoV-2 virus WA strain for 1 hr at 37 °C. The serial dilutions of virus–serum mixtures were then added to Vero E6 cells (ATCC no. CRL-1586) in duplicate wells and incubated for 1 hr at 37 °C with 5% CO2. Cell culture medium with 1% agarose was then added and incubated for three days. The plates were fixed and stained after three days of culture. ID50 and ID90 were calculated as the highest serum dilution resulting in 50 and 90% reduction of plaques, respectively.

### TCID50 assays to measure viral loads

Oral swabs were collected at Day 0, 2, 5 and 8 post SARS-CoV-2 infections. Fourteen days after SARS-CoV-2 infection, all the animals were necropsied, and lung tissue were collected. Oral swabs and lung tissue samples were subjected to viral load measurements. TCID50 assays were used to measure viral loads as described before (7). Vero TMPRSS2 cells (kindly provided by Adrian Creanga from the Vaccine Research Center-NIAID, USA) were plated and cultured in DMEM + 10% FBS + Gentamicin at 37°C, 5.0% CO2. Twenty (20) μL of sample was serially 10-fold diluted and added in quadruplicate to 80 -100% confluent cells and incubated at 37°C, 5.0% CO_2_ for 4 days with 2% of FBS. Virus stock of known infectious titer was included in the assay as a positive control, while medium only served as a negative control. Cytopathic effect (CPE), assessed as cell rounding, in contrast to clear confluent cells, was visually inspected, and was marked as a +, while the absence of CPE was marked as -. The TCID50 value was calculated using the Read-Muench formula.

### Statistical analysis

Statistical analyses were performed using Prism version 9. Area under curve (AUC) values were used for weight loss, clinical scores, ruffled fur scores, hunched back scores, and viral load over time points. Mann-Whitney, ANOVA with multiple comparison corrections, and Spearman analyses were used for group comparisons and correlations as shown in the figures. All statistical tests were 2-tailed, and P were shown in the figures.

## Data availability statement

The original contributions presented in the study are included in the article/**Supplementary Material**. Further inquiries can be directed to the corresponding author.

## Ethics statement

All animal studies were approved by the Institutional Animal Care and Use Committee of BIOQUAL, Inc. and conducted in compliance with all relevant regulations.

## Author contributions

YS and JB designed and interpreted the project. HA, SK, and ML participated in study design and interpreted the experiments. YS prepared the vaccines. SK and HA and their team performed the animal study, PRNT assays, and viral load measurements. YS, JL, TH, and YB performed antibody assays. YS performed statistical analyses. YS and JB wrote the manuscript with input from all the coauthors. All authors contributed to the article and approved the submitted version.
