# Supplementary material for: Protection from COVID-19 disease in hamsters vaccinated with subunit SARS-CoV-2 S1 mucosal vaccines adjuvanted with different adjuvants
Source: Front Immunol. 2023 Mar 20;14:1154496. doi: 10.3389/fimmu.2023.1154496 (PMC10067881; doi:10.3389/fimmu.2023.1154496)

SFig1. Sex difference on weight loss after SARS-CoV-2 Washington strain challenge in hamsters. A-B). Kinetics of body weight change in females and males of difference groups. C-D). Comparisons of Area under curve of body weight changes in females and males. Two-way ANOVA and Mann-Whitney analysis were used to compare between the vaccinated groups and the control group. Mean±SEM are shown.

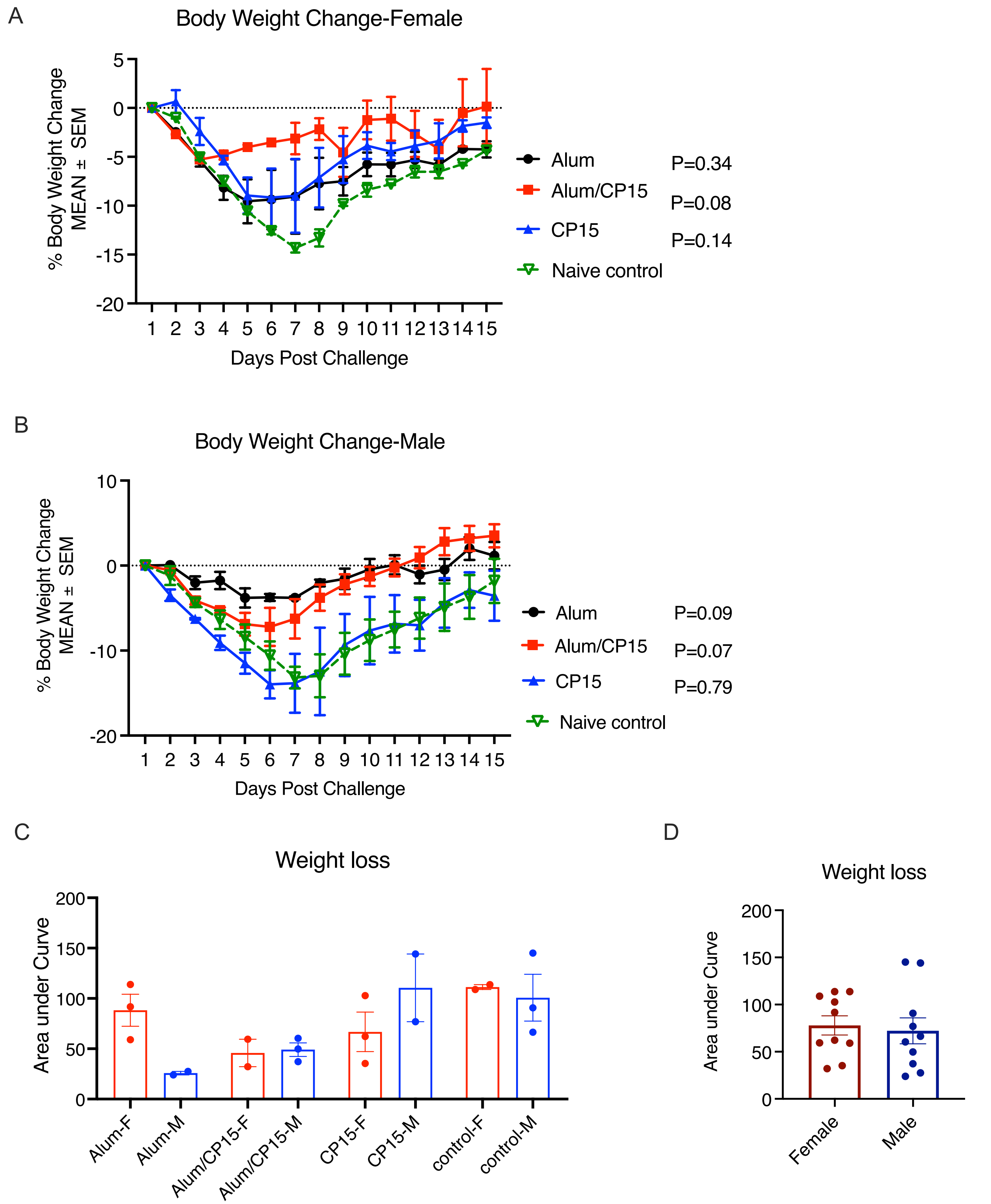

SFig2. Viral load in the lung of vaccinated and naïve animals 14 **days after SARS-CoV-**2 **Washington strain infection.** Geometric means were shown. N=5 for each group, and each dot represents one animal. Dashed line shows the detection limit of the assay.

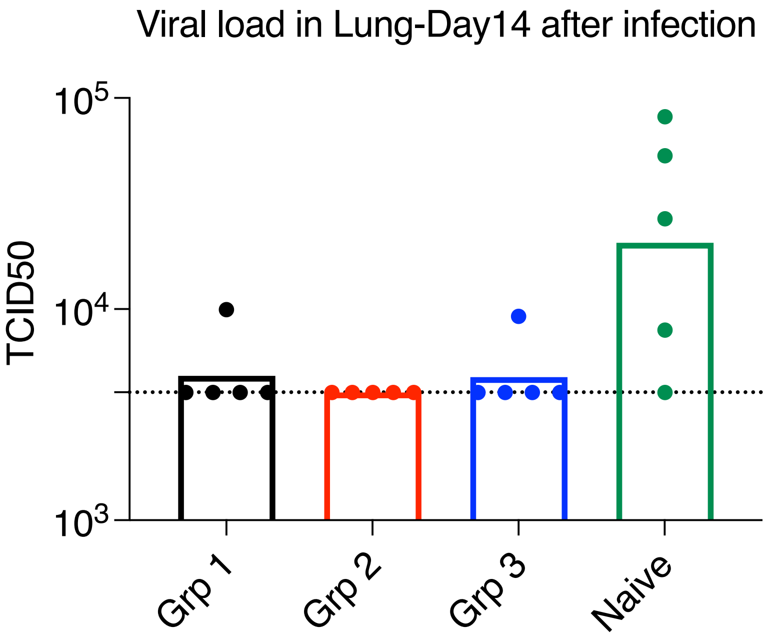

SFig3. Ruffled fur scores of the hamsters after SARS-CoV-2 Washington strain challenge. A-B). Kinetics of ruffled fur score changes in all the animals (A), females(B), and males (C) of difference groups. D-E). Comparisons of Area under curve of ruffled fur score changes in females and males. Triangles denote males, and dots denote females in D. Two-way ANOVA and Mann-Whitney analysis were used to compare between the vaccinated groups and the control group. Mean±SEM are shown.

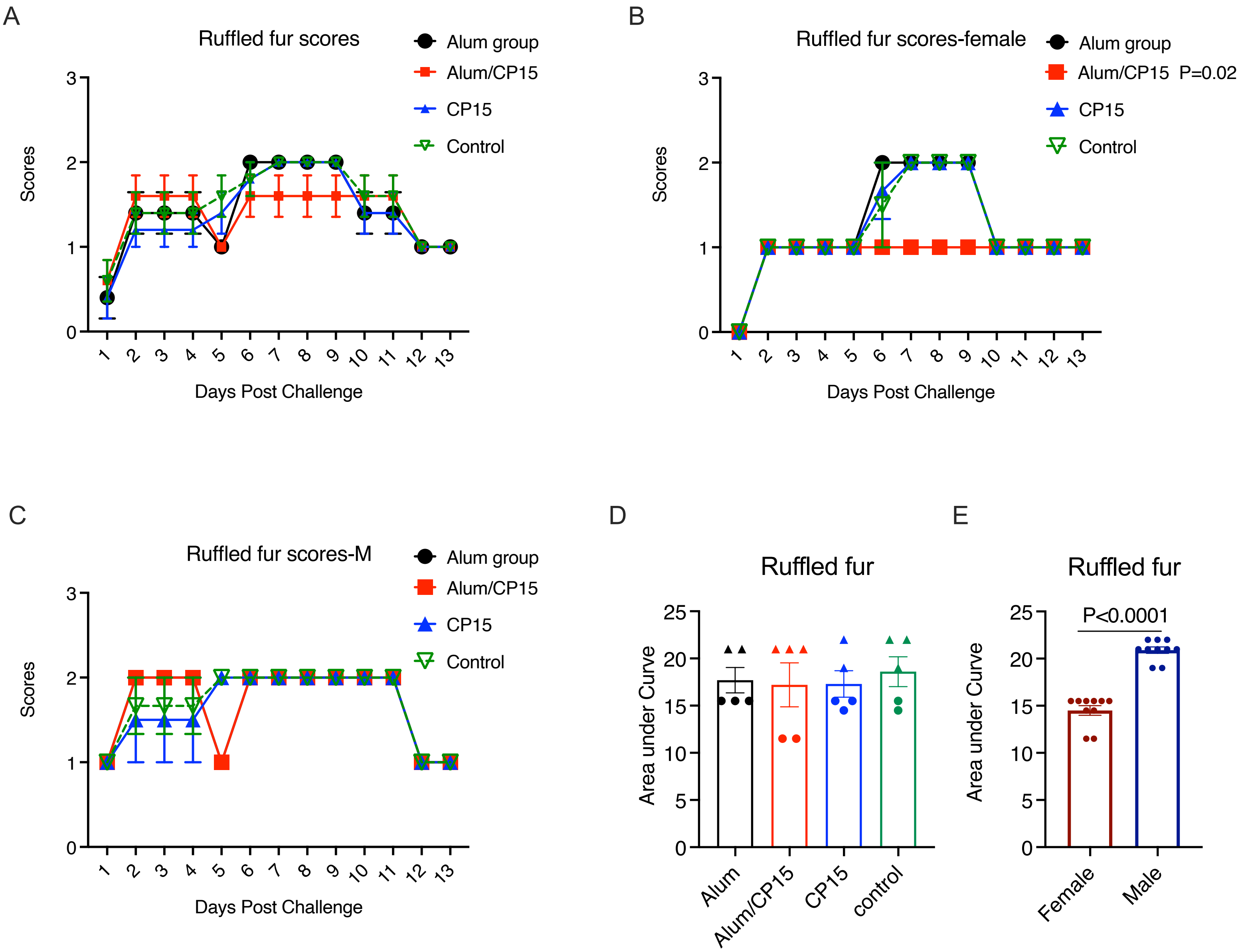

SFig4. Hunched back scores of the hamsters after SARS-CoV-2 Washington strain challenge. A-B). Kinetics of hunched back score changes in all the animals (A), females(B), and males (C) of difference groups. D-E). Comparisons of Area under curve of hunched back score changes in females and males. Triangles denote males, and dots denote females in D Two-way ANOVA and Mann-Whitney analysis were used to compare between the vaccinated groups and the control group. Mean±SEM are shown.

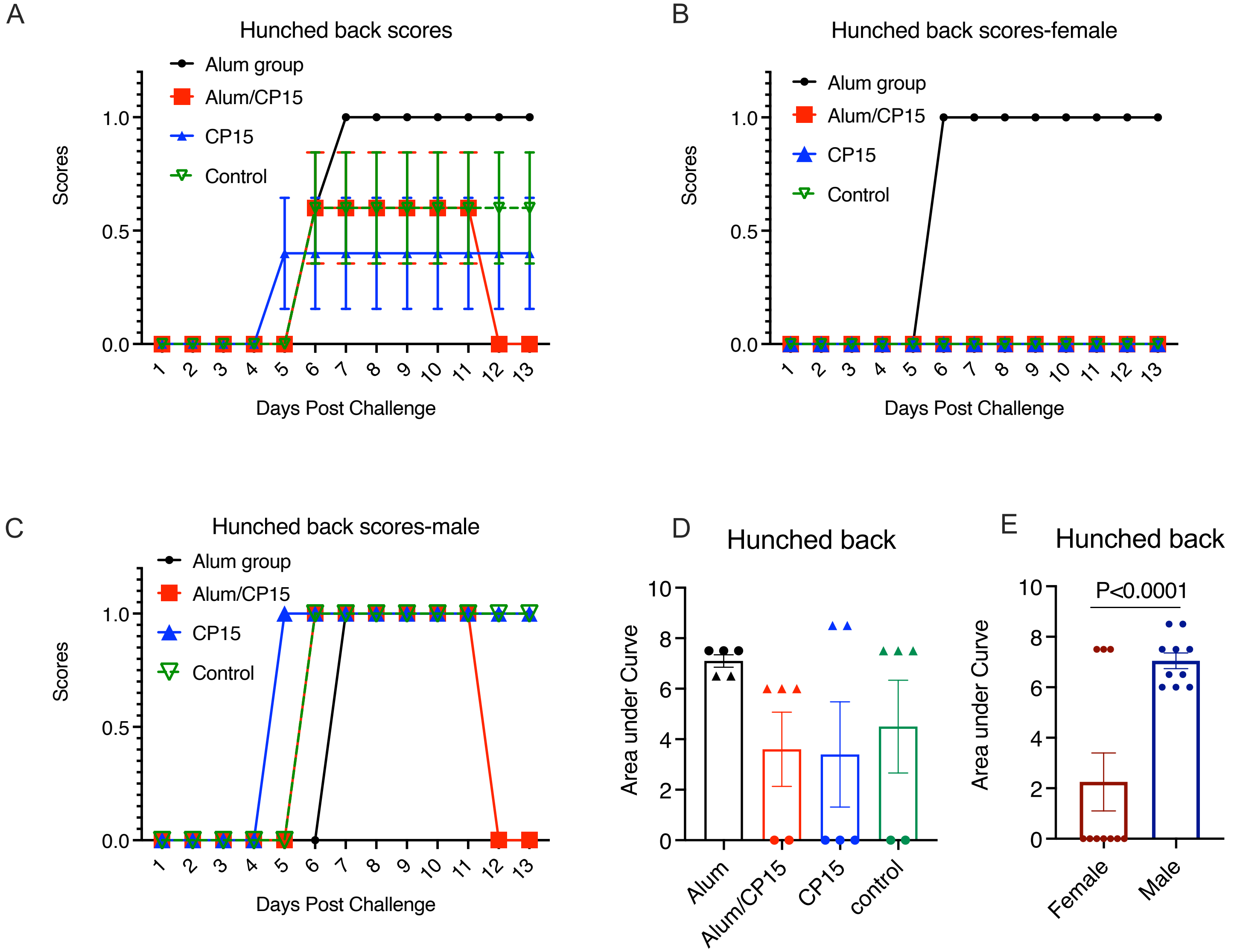

Supplement: Supplementary file 1 [file DataSheet_1.pdf]
